# Chlorogenic Acid Attenuates Isoproterenol Hydrochloride-Induced Cardiac Hypertrophy in AC16 Cells by Inhibiting the Wnt/β-Catenin Signaling Pathway

**DOI:** 10.3390/molecules29040760

**Published:** 2024-02-07

**Authors:** Kai He, Xiaoying Wang, Tingting Li, Yanfei Li, Linlin Ma

**Affiliations:** 1 Graduate School, Shanghai University of Traditional Chinese Medicine, Shanghai 201203, China; 13677306258@163.com (K.H.); wxy1996812@163.com (X.W.); 2 College of Medical Technology, Shanghai University of Medicine and Health Sciences, Shanghai 201318, China; litt@sumhs.edu.cn

**Keywords:** chlorogenic acid, cardiac hypertrophy, Wnt/β-catenin

## Abstract

Cardiac hypertrophy (CH) is an important characteristic in heart failure development. Chlorogenic acid (CGA), a crucial bioactive compound from honeysuckle, is reported to protect against CH. However, its underlying mechanism of action remains incompletely elucidated. Therefore, this study aimed to explore the mechanism underlying the protective effect of CGA on CH. This study established a CH model by stimulating AC16 cells with isoproterenol (Iso). The observed significant decrease in cell surface area, evaluated through fluorescence staining, along with the downregulation of CH-related markers, including atrial natriuretic peptide (ANP), brain natriuretic peptide (BNP), and β-myosin heavy chain (β-MHC) at both mRNA and protein levels, provide compelling evidence of the protective effect of CGA against isoproterenol-induced CH. Mechanistically, CGA induced the expression of glycogen synthase kinase 3β (GSK-3β) while concurrently attenuating the expression of the core protein β-catenin in the Wnt/β-catenin signaling pathway. Furthermore, the experiment utilized the Wnt signaling activator IM-12 to observe its ability to modulate the impact of CGA pretreatment on the development of CH. Using the Gene Expression Omnibus (GEO) database combined with online platforms and tools, this study identified Wnt-related genes influenced by CGA in hypertrophic cardiomyopathy (HCM) and further validated the correlation between CGA and the Wnt/β-catenin signaling pathway in CH. This result provides new insights into the molecular mechanisms underlying the protective effect of CGA against CH, indicating CGA as a promising candidate for the prevention and treatment of heart diseases.

## 1. Introduction

Cardiovascular diseases pose a severe threat to public health [1]. It has been reported that the incidence and mortality rates of cardiovascular diseases worldwide are increasing annually [2]. Heart failure represents the terminal stage of various cardiovascular diseases, with cardiac hypertrophy (CH) being a contributing factor to the onset and mortality of diverse cardiovascular conditions [3]. CH is an adaptive response of the heart to various stimuli and a crucial process in the development of various cardiovascular diseases leading to heart failure [4,5]. In response to adverse stimuli, cardiomyocytes exhibit increased protein synthesis, cardiomyocyte volume, and fibrosis, with pathological manifestations including cardiomyocyte fibrosis, extracellular matrix deposition, and cardiomyocyte hypertrophy [6,7]. Research indicates that certain cardiac developmental transcription factors exhibit upregulation and/or increased transcriptional activity in response to hypertrophic stimuli, which leads to the elevated expression of downstream cardiac embryonic genes such as those coding for atrial natriuretic peptide (ANP) and brain natriuretic peptide (BNP) and the subtype conversion of myosin heavy chain (MHC) [8,9,10,11,12,13]. This phase represents an adaptive compensatory response under conditions of pressure overload, but disease progression may lead to ventricular enlargement, impaired cardiac function, and adverse cardiovascular outcomes [14,15]. Prolonged CH typically occurs before the development of heart failure, but research on therapeutic strategies to prevent CH is still limited, and the actual treatment plan remains to be explored [6,16].

The Wnt signaling pathway plays a significant role in the differentiation of cardiomyocytes in the heart, being involved in regulating metabolism, immune responses, and the development of various cancers [17,18]. Reports indicate pivotal roles of the Wnt signaling pathway in CH and ventricular remodeling [19,20]. Improper activation of the Wnt/β-catenin pathway in adulthood is closely associated with various cardiac conditions such as dilated cardiomyopathy [21], hypertensive heart disease [22], and myocardial infarction [23]. Wnt pathway expression in cardiac myocytes is regulated by a complex involving Frizzled (Fzd) binding to the low-density lipoprotein receptor-related protein 5/6 (LRP5/6) complex and Wnt ligands, which modulates target gene expression through the regulation of Wnt protein synthesis [24,25]. In the Wnt/β-catenin signaling pathway, the activation of β-catenin leads to pathological hypertrophy in cardiomyocytes [7,19,26]. Studies indicate that the activation of the Wnt/β-catenin signaling pathway is involved in the negative regulation of cardiovascular diseases [27,28,29]. The activation of the Wnt/β-catenin pathway triggers the expression of hypertrophy-related genes in cardiomyocytes, leading to cardiac dysfunction [25,30]. Glycogen synthase kinase-3β (GSK-3β) is one of the negative regulatory factors that can counteract hypertrophic responses, and inhibition of GSK-3β results in an increase in the quantity of β-catenin protein [31,32]. Meanwhile, the interruption of Wnt signaling, involving GSK-3β, can mitigate pressure overload-induced CH [33]. Therefore, modulating the Wnt/β-catenin signaling pathway may contribute to treating myocardial diseases.

The traditional Chinese herbal medicine honeysuckle, historically recognized for its heat-clearing and detoxifying properties, contains key active compounds such as chlorogenic acid (CGA), isochlorogenic acid, and caffeic acid [34,35,36]. CGA, formed by one molecule of caffeic acid and one molecule of quinic acid, contains ester bonds, unsaturated double bonds, and two unstable phenolic groups in its molecular structure, which makes it susceptible to oxidation and hydrolysis under high temperatures [37,38]. According to research, the primary active component of honeysuckle is chlorogenic acid [39,40]. With a molecular formula of C16H18O9, CGA is renowned for its anti-apoptotic [38], antioxidant [38], anti-inflammatory [38], and analgesic effects [41]. Currently, research on the pathogenesis of CH focuses on aspects such as excessive cardiac pressure load, cardiomyocyte apoptosis, self-vascular remodeling, inflammatory response, and oxidative stress response [42]. Quantitative results regarding the myocardial cell surface area and the mRNA expression of molecular markers associated with CH indicated the potential cardiovascular protective effects of CGA [43]. Additionally, research indicates that CGA, when used for the prevention and/or treatment of liver damage, exhibits extremely low toxicity, ensuring medication safety, and based on animal safety tests (long-term toxicity tests), the estimated safe dose for human use is not greater than 90 mg/kg, corresponding to a daily dose not exceeding 4500 mg (for a weight of 50 kg) [44]. The pharmacokinetic parameters of CGA may vary significantly across different formulations, doses, and administration routes [45]. A study reported that the measured blood drug concentrations in rats after the administration of CGA at doses of 20 mg/kg and 80 mg/kg revealed that Cmax was reached approximately 30 min after treatment, with both the half-life and the AUC increasing as the dose increased [46]. Moreover, research indicated that the metabolism of CGA, following oral administration in both animals and humans, is primarily due to the intestinal microbiota [45,47]. Nevertheless, the mechanisms underlying the action of CGA in CH have not been completely clarified.

Therefore, based on the crucial role of the Wnt/β-catenin signaling pathway in cardiomyocytes, this study utilized isoproterenol hydrochloride (Iso) to induce CH in AC16 cells, aiming to investigate whether CGA is involved in the regulation of the Wnt/β-catenin signaling pathway, thereby inhibiting the development of CH.

## 2. Results

### 2.1. Effects of CGA on the Viability of AC16 Cells

**Figure 1:**
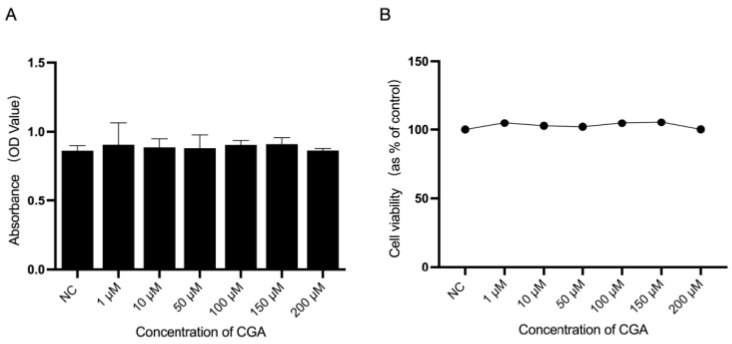
Effects of CGA on the viability of AC16 cells. (**A**) The optical density values of AC16 cells changed with increasing CGA concentrations. (**B**) Viability of AC16 cells treated with different concentrations of CGA. The survival rate of AC16 cells in the NC group was set to 100, and the data in the line graph represent cell viability after the different treatments as a percentage of that of the NC group. Data are the mean ± SD. ns *p* > 0.05 compared to the NC group. NC, negative control; CGA, chlorogenic acid.

To evaluate the effects of CGA on AC16 cells, we used a CCK-8 assay kit to measure cell viability, which determines the survival rate and activity of cells based on their optical density (OD) values. The results showed that, compared with the NC group, even within the concentration range of 1–200 µM, CGA did not significantly affect the viability of AC16 cells measured by the CCK-8 assay (*p* > 0.05; Figure 1). This conclusion was based on the similar optical density values observed for the experimental and the control groups. Building upon this foundation and integrating experimental results from previous relevant studies [48,49,50], a model concentration of 150 μM for CGA was chosen as the highest concentration for the experimental setup in the later stages of this research.

### 2.2. Effect of CGA on the Surface Area of AC16 Cells

**Figure 2:**
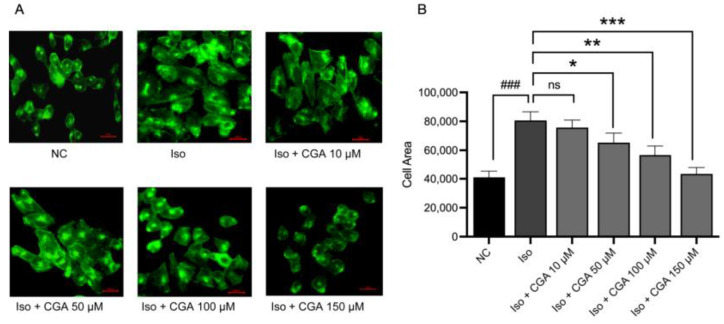
Effect of CGA on the surface area of AC16 cells. (**A**) Changes in the size of AC16 cells were captured after treatment with different concentrations of CGA or/and Iso (10 µM). (**B**) The area of AC16 cells was quantified using ImageJ software (version 1.83). ### *p*< 0.001 vs. NC group, ns *p* > 0.05, * *p* < 0.05, ** *p* < 0.01, *** *p* < 0.001 vs. the Iso group. NC, negative control; CGA, chlorogenic acid; Iso, isoproterenol.

To examine the effect of CGA pretreatment on the progression of CH in AC16 cells, fluorescence microscopy was used to observe changes in AC16 cardiomyocytes. The results showed that compared with the Iso group, myocardial cells pretreated with different concentrations of CGA had a reduced cell surface area (*p* < 0.001; Figure 2B). These findings suggest that CGA pretreatment might contribute to the prevention of CH in AC16 cells; the greatest effect was seen at a concentration of 150 µM (*p* < 0.001; Figure 2A,B).

### 2.3. Effect of CGA on the Transcription of ANP, BNP, and β-MHC in AC16 Cells

**Figure 3:**
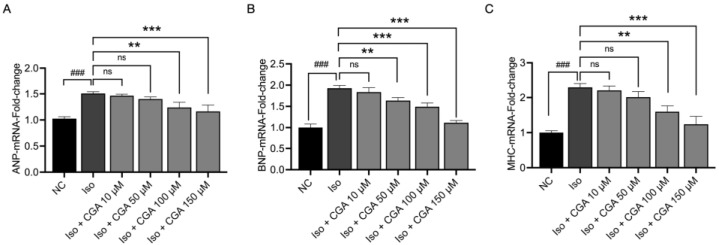
Effects of CGA on the transcriptional expression of ANP, BNP, and β-MHC in AC16 cells. (**A**–**C**) mRNA expression of the hypertrophy molecular markers ANP (**A**), BNP (**B**), and β-MHC (**C**), determined by qPCR. Data are presented as mean ± standard deviation. ### *p* < 0.001 compared to the NC group, ns *p* > 0.05, ** *p* < 0.01, *** *p* < 0.001 compared to the Iso group.

Based on the above results, this study further determined the changes in the mRNA expression levels of three hypertrophy molecular markers, i.e., ANP, BNP, and β-MHC, to elucidate the impact of CGA pretreatment on Iso-induced hypertrophy in AC16 cells (Figure 3). The results indicated that, compared to the NC group, the mRNA expression levels of ANP, BNP, and β-MHC significantly increased in Iso-induced cardiac myocytes (*p* < 0.001; Figure 3A–C). In contrast, the group pretreated with CGA exhibited a significant inhibition of the Iso-induced increase in the ANP, BNP, and β-MHC expression levels (*p* < 0.001; Figure 3A–C). This study presents the successful establishment of an in vitro model of CH induced by Iso, showing that CGA pretreatment effectively reduced the changes in the expression of hypertrophic markers induced by Iso. Therefore, these results provide evidence supporting the potential use of CGA as a preventive measure for CH in AC16 cells.

### 2.4. Effect of CGA on the Protein Expression Levels of ANP and BNP in AC16 Cells

**Figure 4:**
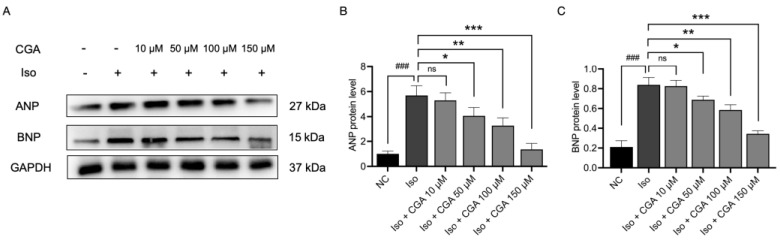
Effect of CGA on the protein expression levels of ANP and BNP in AC16 cells. (**A**) Protein expression of ANP and BNP was measured by WB and statistically analyzed (**B**,**C**). Data are presented as mean ± SD. ### *p* < 0.001 vs. the NC group, ns *p* > 0.05, * *p* < 0.05, ** *p* < 0.01, *** *p* < 0.001 vs. the Iso group. NC, negative control; CGA, chlorogenic acid; Iso, isoproterenol; ANP, atrial natriuretic peptide; BNP, brain natriuretic peptide.

The results showed that, compared to the NC group, the protein expression levels of ANP and BNP in AC16 cells significantly increased after 24 h of induction with 10 µM Iso (*p* < 0.001; Figure 3A–C). However, after pretreatment with CGA, the protein expression levels of ANP and BNP in AC16 cells were significantly lower than those in the Iso group (*p* < 0.001; Figure 4). These results suggest that the effect of CGA in Iso-induced AC16 cells may be concentration-dependent, with the most significant effect observed at a concentration of 150 µM (Figure 4). These findings indicate a potential role of CGA in preventing CH.

### 2.5. Effect of CGA on the Wnt/β-Catenin Signaling Pathway

**Figure 5:**
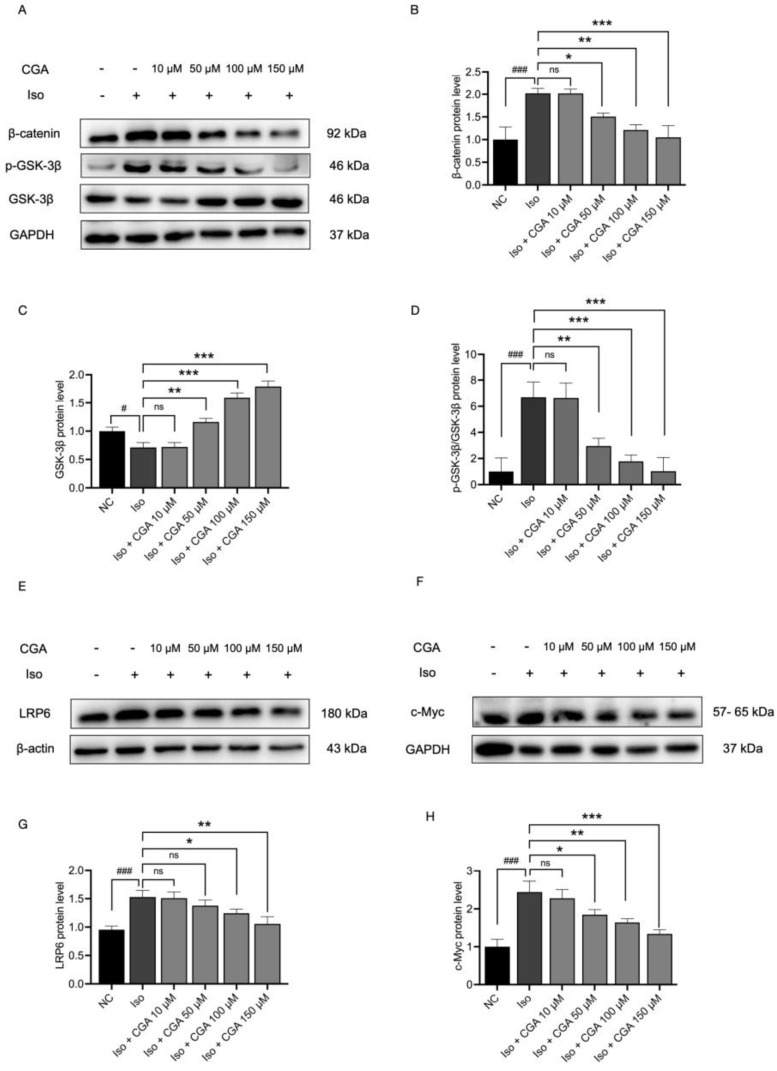
Effect of CGA on the Wnt/β-catenin signaling pathway. (**A**,**E**,**F**) Changes in the protein levels of Wnt/β-catenin signaling pathway markers, including β-catenin, GSK-3β, p-GSK-3β/GSK-3β, LRP6, and c-Myc, were detected by Western blotting in AC16 cells pretreated with various concentrations of CGA or/and Iso (10 μM). (**B**−**D**,**G**,**H**) Changes in β-catenin, GSK-3β, p-GSK-3β/GSK-3β, LRP6, and c-Myc expression quantified by ImageJ software (version 1.83). Data are presented as mean ± SD. # *p* < 0.05, ### *p* < 0.001 vs. control group, ns *p* > 0.05, * *p* < 0.05, ** *p* < 0.01, *** *p* < 0.001 vs. Iso group. NC, negative control; CGA, chlorogenic acid; Iso, isoproterenol; LRP6, low-density lipoprotein receptor-related protein 6; p-, phosphorylated; GSK3β, glycogen synthase kinase 3β.

To further investigate the potential mechanism by which CGA inhibits Iso-induced hypertrophy in AC16 cells and, particularly, to validate whether it involves the regulation of the Wnt/β-catenin signaling pathway, this experiment evaluated the expression of the relevant protein components of this pathway. In comparison to the NC group, the Iso group exhibited a significant decrease in the expression level of GSK-3β (*p* < 0.05; Figure 5A,C), while the protein expression levels of LRP6, β-catenin, and c-Myc and the expression ratio of p-GSK-3β to total GSK-3β significantly increased (*p* < 0.05; Figure 5A–F). Following pretreatment with varying concentrations of CGA, the protein expression levels of LRP6, β-catenin, and c-Myc and the expression ratio of p-GSK-3β to total GSK-3β showed a gradual decreasing trend (*p* < 0.05; Figure 5B,D,G,H), while GSK-3β expression gradually increased (*p* < 0.05; Figure 5A,C).

### 2.6. Effect of CGA and IM-12 on the Expression of Proteins in the Wnt Signaling Pathway

**Figure 6:**
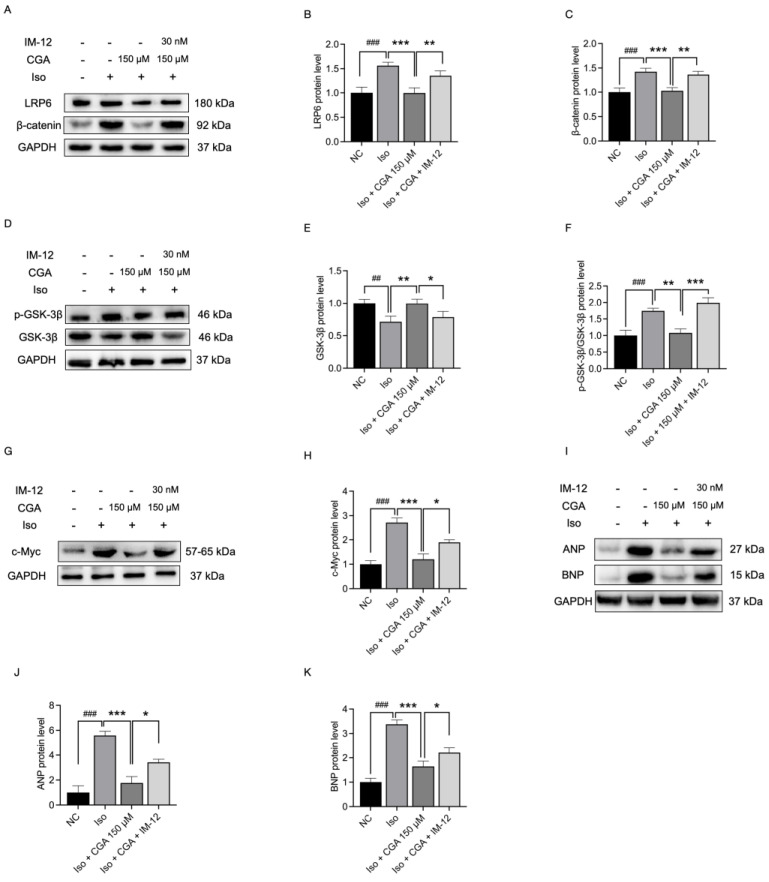
Effect of CGA and IM-12 on the expression of proteins in the Wnt signaling pathway. (**A**,**D**,**G**,**I**) Changes in the protein levels of Wnt/β-catenin signaling pathway markers, including LRP6, β-catenin, GSK-3β, p-GSK-3β/GSK-3β, c-Myc, ANP, and BNP. (**B**,**C**,**E**,**F**,**H**,**I**−**K**) Changes in LRP6, β-catenin, GSK-3β, p-GSK-3β/GSK-3β, c-Myc, ANP, and BNP expression quantified by ImageJ sofware (version 1.83). Data are presented as mean ± SD. ## *p* < 0.01, ### *p* < 0.001 vs. the NC group, ns *p* > 0.05, * *p* < 0.05, ** *p* < 0.01, *** *p* < 0.001 vs. Iso group/vs. control group. NC, negative control; CGA, chlorogenic acid; Iso, isoproterenol; LRP6, low-density lipoprotein receptor-related protein 6; p-, phosphorylated; GSK3β, glycogen synthase kinase 3β; ANP, atrial natriuretic peptide; BNP, brain natriuretic peptide.

To further confirm that CGA inhibits the development of CH by modulating the Wnt/β-catenin signaling pathway, this study employed the Wnt activator IM-12 to investigate the relationship between CGA inhibition of CH and Wnt/β-catenin signaling pathway activity. According to research reports, IM-12 enhances Wnt signaling by inhibiting GSK-3β [51]. Similar to previous research findings, pretreatment with 150 μM CGA resulted in a significant increase in GSK-3β protein expression (*p* < 0.01; Figure 6D,E), while the protein expression levels of LRP6, β-catenin, and c-Myc, and the expression ratio of p-GSK-3β to total GSK-3β showed a significant decrease (*p* < 0.001; Figure 6A–H). However, in comparison to the CGA preventive group, the CGA + Iso + IM-12 group exhibited a significant increase in the protein levels of LRP6, β-catenin, and c-Myc and in the expression ratio of p-GSK-3β to total GSK-3β (*p* < 0.01), as well as the decrease in GSK-3β (*p* < 0.05) protein expression levels. Additionally, the study observed a partial reversal of the CGA-mediated inhibition of the expression of the CH molecular markers ANP (*p* < 0.001) and BNP (*p* < 0.05) in the CGA + Iso + IM-12 group (Figure 6I−K). These results validated that CGA may mediate its inhibitory effect on CH through the Wnt/β-catenin signaling pathway.

### 2.7. Identification of DEGs in HCM Tissue Compared to Healthy Tissue

**Figure 7:**
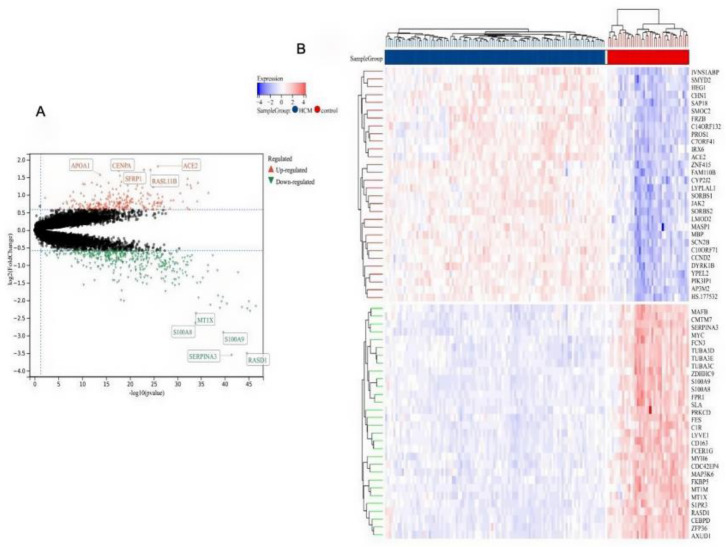
Identification of differentially expressed genes (DEGs) in HCM tissue. (**A**) Volcano plots of DEGs in HCM tissue. (**B**) Heatmaps of the top 30 DEGs in HCM tissue. Red, up-regulated DEGs; blue, down-regulated DEGs.

The human cardiac tissue expression dataset GSE36961 (106 HCM samples and 39 controls) was used for our differential gene expression analysis. After data preprocessing and differential expression analysis, we found 639 DEGs between the HCM samples and the healthy controls (|fold change| ≥ 1.5, and *p* value < 0.05), including 249 up-regulated and 390 down-regulated genes. The genes with the highest expression differences were ACE2, APOA1, CENPA, SFRP1, RASL11B, RASD1, SERPINA3, S100A9, S100A8, and MT1X (Figure 7A). The top 30 DEGs in the HCM samples (Figure 7B), including IVNS1ABP, SMYD2, HEG1, CHN1, SAP18, MAFB, CMTM7, SERPINA3, MYC, and FCN3, are shown in a heatmap (Figure 7B). 

### 2.8. WGCNA of the Whole Transcriptome Expression Matrix 

**Figure 8:**
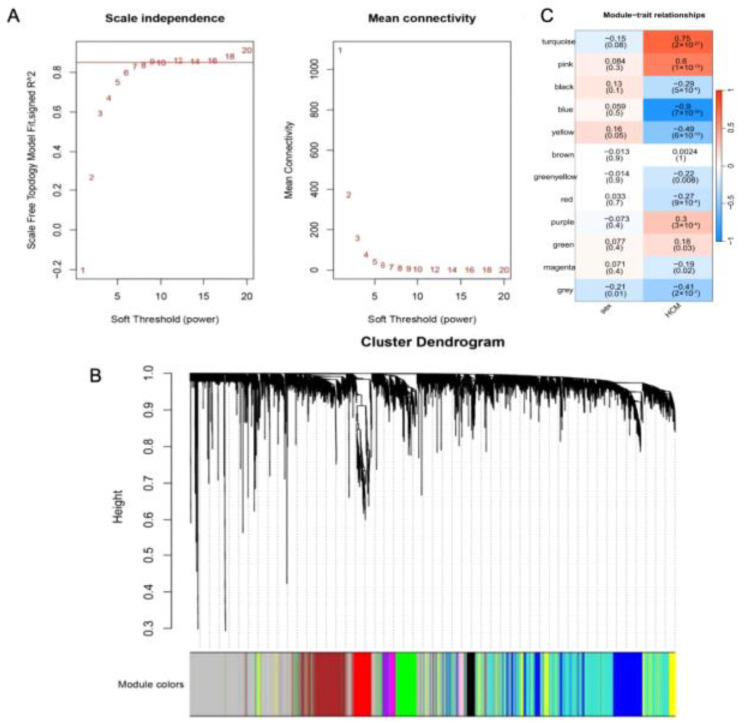
Identification of modules correlated with HCM in transcriptome datasets. (**A**) The purpose of the analysis was to evaluate the independence of scale and the average connectivity for the best soft-thresholding powers in the HCM dataset. (**B**) The samples in the HCM dataset were used to construct hierarchical clustering dendrograms. (**C**) Diagrams illustrating the relationship between modules and traits were also created for the HCM dataset, where each row represents a color module, and each column represents a clinical trait. The correlation and *p*-value for each combination of module and trait are shown in the corresponding cells.

The WGCNA analysis was carried out to uncover gene expression patterns with similar biological functions in the GSE36961 dataset. The optimal soft-thresholding parameters were determined by examining the network topology, with a β value of 9 being identified as the most suitable (Figure 8A). A total of 11 modules were identified from the dataset (Figure 8B). Module–trait diagrams were constructed to investigate the connections between gene modules and HCM. Of these, the MEblue (r = −0.9, *p* = 7 × 10^-55^), MEturquoise (r = 0.75, *p* = 2 × 10 ^-27^), and MEpink (r = 0.6, *p* = 1 × 10^-15^) modules exhibited the highest correlation to HCM (Figure 8C).

### 2.9. Determination of Wnt-Related Genes Targeted by CGA in HCM-Affected Cells and Functional Enrichment Analysis

**Figure 9:**
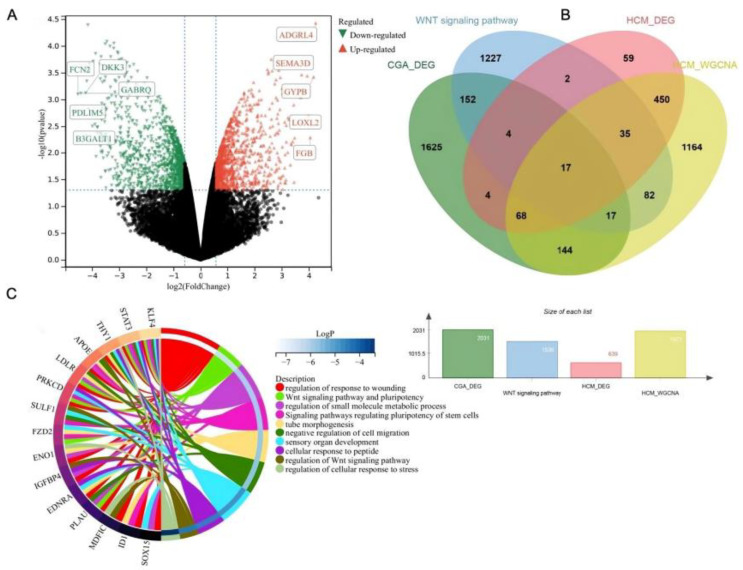
Functional enrichment analysis of Wnt signaling pathway-related targets of CGA in HCM-affected cells. (**A**) Volcano plots of DEGs associated with CGA. (**B**) Venn diagram analysis of the targets of CGA in HCM-affected cells involving the Wnt signaling pathway. (**C**) The top 10 enriched functional terms for 17 intersecting targets.

To identify the Wnt-signaling pathway-related genes targeted by CGA in HCM-affected cells, a Venn analysis was performed on the DEGs associated with CGA, Wnt signaling pathway-related genes, the HCM DEGs, and hub genes in WGCNA. As shown in Figure 9A, 2031 genes were identified in GSE85871. Then, 1536 genes were retrieved from GeneCards (score > 1). A total of 17 intersecting genes were obtained using the above-mentioned four genesets and were imported into the Metascape database to carry out functional enrichment analysis (Figure 9B). In addition to the Wnt signaling pathway, other important terms included regulation of response to wounding, regulation of small molecule metabolic process, signaling pathways regulating pluripotency of stem cells, tube morphogenesis, and negative regulation of cell migration, including several hub genes (KLF4, STAT3, THY1, APOE, LDLR, PRKCD, SULF1, FZD2, ENO1, IGFBP4) (Figure 9C).

## 3. Discussion

Cardiomyocytes in the adult heart may nearly lose their ability to proliferate, and myocardial damage might be irreversible [52,53]. The signaling mechanisms that trigger CH are complex, and current studies focus on inflammation and oxidative stress [54,55]. Dysregulation of the Wnt/β-catenin pathway plays a crucial role in the pathogenesis of CH [56,57]. The Wnt signaling pathway was also reported to be associated with heart disease; overstimulation of Wnt signaling is detrimental to cardiovascular pathology [58]. CGA, with multiple pharmacological effects including anti-inflammatory and anti-apoptotic effects, is a promising cardioprotective agent that can inhibit the development of CH [59,60]. Recent studies indicated that CGA exerts a regulatory effect on the Wnt/β-catenin signaling pathway in colorectal cancer cells [61]. Furthermore, CGA can suppress epithelial–mesenchymal transition and invasion in breast cancer by downregulating the expression of LRP6, a component of the Wnt/β-catenin signaling pathway [62]. Moreover, Iso is a synthetic β-adrenergic agonist known to induce myocardial stress, subsequently leading to CH [63]. These findings may offer a viable approach and strategy for mitigating CH.

Therefore, in this study, the AC16 cell line treated with Iso was employed to establish a model of CH. According to the results of the CCK-8 assays (Figure 1), different concentrations of CGA had a small effect on the viability of cardiomyocytes. Studies reported that the early stage of CH is characterized by an increase in the surface area of cardiomyocytes, and changes in the surface area of cardiomyocytes can directly reflect the development of CH [64,65] (quantitative analysis using immunofluorescence) (Figure 2). The mRNA and protein expression of ANP, BNP, and β-MHC, considered molecular markers of CH, is significantly increased in heart failure [66]. In this study, CGA reduced the expression of the CH markers ANP and BNP in a dose-dependent manner, with the CGA concentration of 150 µM identified as the optimal one in relation to mRNA expression. Additionally, our study found that CGA at 50 µM did not downregulate ANP mRNA levels but did decrease ANP protein expression. Previous research showed a trend in ANP mRNA changes after treatment with CGA 50 µM without statistical significance [43], consistent with our experimental results (Figure 3), while the changes in the protein expression levels of ANP demonstrated statistical significance (Figure 4). This suggests that the mechanism of action of CGA may involve the reduction of ANP protein expression, although the specific underlying mechanism requires further investigation. In summary, CGA pretreatment in this study demonstrated a protective effect against Iso-induced CH, as evidenced by the reduced cardiomyocyte area and expression of CH markers (Figure 3 and Figure 4).

Due to the important role of CGA and the Wnt/β-catenin signaling pathway in CH, myocardial fibrosis, heart failure, and other cardiovascular diseases [67,68], their interplay holds considerable importance in these contexts. In our study, we further investigated whether pre-treatment with CGA attenuated Iso-induced CH by modulating the Wnt/β-catenin signaling pathway. Different Wnt ligands activate different intracellular transduction signaling pathways [69,70]. When Wnt ligands are present, the Wnt/β-catenin pathway is activated; in fact, extracellular Wnt proteins, Frz receptor membrane proteins, and the low-density lipoprotein-related receptors LRP5/6 bind and activate Wnt/β-catenin signaling, leading to the activation of the Dsh protein in the cytoplasm and promoting the phosphorylation of GSK-3β [71]. Therefore, β-catenin cannot be recognized and degraded by the ubiquitin protease system, and non-phosphorylated β-catenin is accumulated in the cytoplasm, which results in the promotion of β-catenin binding to TCF/LEF transcription factors in the nucleus and the activation of downstream target genes such as c-Myc. This stimulates cell proliferation and increases cell resistance to apoptosis [72,73,74].

Based on this, the Western blotting analysis in this study provides evidence for a potential role of CGA in the inhibition of the Wnt/β-catenin signaling pathway during the development of CH. As shown in this study, CGA induced changes in the expression of GSK-3β and β-catenin. It was observed that in the Iso group, LRP6, β-catenin, Wnt5a/b, and c-Myc were significantly up-regulated compared with the control group, but significantly down-regulated after pretreatment with CGA (Figure 5). Interestingly, the expression level of GSK-3β protein showed an opposite trend after Iso stimulation, which we inferred might be related to its negative regulation of the Wnt pathway and indicated that the activity of GSK-3β was blocked after Wnt signaling activation [75]. Research showed that a reduction in GSK-3β expression in cardiac fibroblasts led to adverse ventricular remodeling [76]. Conversely, the absence of β-catenin does not affect the structural integrity of cardiac muscle cells, and as the levels of β-catenin increase, so do the levels of proteins associated with CH [77,78]. Therefore, the inhibition of GSK-3β and the accumulation of β-catenin may increase the activity of Wnt signaling, leading to CH. These above observations confirm previous studies, suggesting that the protective effect of CGA on CH may be mediated through the Wnt/β-catenin signaling pathway during the development of CH.

To verify the above conjecture, we used in this study IM-12, a specific agonist of the Wnt signaling pathway that was reported to specifically inhibit the expression of GSK-3β while increasing the expression of β-catenin and downstream proteins [51,79,80]. In this study, it was observed that pre-treatment with CGA partially suppressed the expression of core proteins associated with the Wnt signaling pathway activated by IM-12, such as β-catenin, p-GSK-3β, and c-Myc, while concurrently partially increasing the expression of GSK-3β (Figure 6). This suggests that CGA may engage in the regulation of CH development by modulating the Wnt signaling pathway.

To further investigate the correlation between CGA and the Wnt/β-catenin signaling pathway in CH, the datasets GSE36961 and GSE85871 were selected from the GEO database for preprocessing and differential expression analysis (Figure 7A, B). These datasets were also utilized for WGCNA, leading to module–trait relationship diagrams and the compilation of gene lists associated with each module (Figure 8A–C). The identification of Wnt-related genes influenced by CGA in HCM tissue was performed using a free online platform, and functional enrichment analysis revealed multiple signaling pathways beyond the Wnt signaling pathway (Figure 9A–C). These findings illustrated that CGA can modulate the development of CH by influencing the Wnt/β-catenin signaling pathway.

However, it should be noted that, in our study, only core proteins in the Wnt signaling pathway were explored. The detailed underlying molecular mechanisms governing the upstream regulation of the Wnt/β-catenin signaling pathway during CH remain incompletely elucidated. Furthermore, considering the involvement of Wnt signal transduction in the nuclear localization of β-catenin [81], it is equally imperative to determine the localization and expression of β-catenin. Additionally, this study did not delve deeply into the detailed effects of CGA on the upstream and downstream proteins of the Wnt/β-catenin signaling pathway, nor did it investigate the specific phosphorylation sites in GSK-3β. These aspects will need to be addressed in future research endeavors to augment the potential of our results for clinical translation. In future studies, the elucidation of the inhibition-like mechanism of CGA and its impact on other diseases caused by abnormal activation of the Wnt signaling pathway, such as cancer [82], would provide valuable insights into the potential therapeutic effects of CGA in these diseases.

## 4. Materials and Methods

### 4.1. Chemicals, Reagents, and Kits

Iso (CS-2582) was purchased from MCE (Med Chem Express), and CGA (C109404) was purchased from United States, Med Chem Express, Inc., Ltd (Princeton, NJ, United States) (HY-N0055) (isolated purity ≥ 99.5; CGA ≥ 99.5). IM-12 was purchased from United States, Med Chem Express, Inc. The anti-ANP antibody (ab189921) was purchased from Abcam, Inc. (Cambridge, United Kingdom) , and the anti-β-catenin (cat. no. 8480), anti-c-Myc (cat. no. 5605), anti-LRP6 (cat. no. 3395), anti-glycogen synthase kinase-3β (GSK-3β; cat. no. 12456), and anti-p-GSK-3β (cat. no. 9322) antibodies to analyze the Wnt/β-catenin signaling pathway were purchased from United States, Cell Signaling, Inc., Ltd (Beverl, MA, United States). The bicinchoninic acid (BCA) protein quantification kit (P0010) and the Actin-Tracker Green kit (C1033) were purchased from China, Beyotime Biotechnology, Co., Ltd (Shanghai, China). The anti-GAPDH antibody (BM1623) and the horseradish peroxidase-conjugated secondary antibody (BA1054; BM2002) were purchased from China, Wuhan Boster Biotechnology Co., Ltd (Wuhan, China). The RIPA lysis buffer (P0013) was purchased from Beyotime Biotechnology, Ltd (Shanghai, China). The Cell Counting Kit-8 (40203ES76) was purchased from China, Yeasen Biotechnology, Co., Ltd (Shanghai, China).

### 4.2. Culture of AC16 Cells

AC16 cells [83] were cultured in DMEM high-glucose medium containing 10% fetal bovine serum and 1 × 10^5^ U/L of penicillin–streptomycin in a 37 °C, 5% CO_2_ incubator, and the medium was changed every 2 to 3 days. The cells were passaged when well-grown cells in the logarithmic growth phase were used for the experiments.

### 4.3. In Vitro CH Model and Drug Treatment

The cells were randomly divided as follows: (i) Control (NC), cells treated with DMEM; (ii) Iso, cells treated with 10 µM Iso for 24 h; (iii) CGA, cells treated with10 µM, 50 µM, 100 µM, or 150 µM CGA for 6 h, followed by 10 µM Iso for 24 h; (iv) IM-12 + Iso + CGA 150 µM, cells treated with 150 µM CGA for 6 h followed by 10 µM Iso for 24 h and 30 nM IM-12 for 6 h; (v) Iso + CGA 150 µM, cells treated with 150 µM CGA for 6 h followed by 10 µM Iso for 24 h. A total of 1.5–2 × 10^5^ cells were plated in culture flasks with DMEM at 37 °C in 5% CO_2_ and in a humidified atmosphere. The cells were washed three times with PBS before the addition of each drug treatment component to avoid mixing different drugs.

### 4.4. CCK-8 Assay

After trypsinization, the cardiomyocytes were resuspended and plated in 96-well plates. After 24 h, the cells were divided into three groups: control, Iso, and 10, 50, 100, 150, and 200 µmol/L CGA treatment groups. After treatment for 24 h, the cell supernatant was aspirated and discarded, a complete-medium solution containing 10% CCK-8 was added to the culture for 3 h, and the absorbance was detected at a wavelength of 450 nm. A line chart was constructed by plotting the optical density (OD) values against the drug concentrations to assess the effect of CGA on cell viability.

### 4.5. Cell Morphometric Analysis

The concentrations of CGA used in the experiments were 0, 10, 50, 100, and 150 µmol/L. The cardiomyocytes were detached with trypsin, resuspended, and plated in a 6-well plate. After 24 h, the cell culture medium was changed. AC16 cardiomyocytes were pretreated for 6 h according to the set concentration and then stimulated with Iso for 24 h. Actin-Tracker Green was diluted 1:100 with 0.1% Triton and 5% BSA according to the manufacturer’s instructions and then added to each well of the plates, which were wrapped in light-proof tin foil and kept at room temperature. After incubating for 20–60 min, the cells were washed three times with PBS containing 0.1% Triton X-100 by immersing them for 5 min. Under inverted-fluorescence electron microscopy, appropriate magnifications and fields of view were selected to take pictures, and the surface area of myocardial cells in each experimental group was calculated.

### 4.6. Quantitative Reverse Transcription–Polymerase Chain Reaction (qPCR)

The mRNA expression levels of the genes were measured using RT-PCR. Total RNA was extracted from AC16 cells using the Trizol (Sinopharm Chemical Reagent Co., Ltd. Shanghai, China) reagent. We measured RNA concentration and quality using a Nano2000 ultra-micro spectrophotometer (Megu Molecules, Inc. Shanghai, China) to ensure absorbance ratio between 1.8 and 2.0 and obtained cDNA for RT-PCR using 1 µg of total RNA and the Transcript^®^ Green Two-Step RT-PCR Super Mix (AQ201) kit (TransGen Biotech Co., Ltd, Beijing China). The primers (Table 1) were synthesized by Sangon Biotech (Shanghai, China). The specific PCR conditions involved denaturation at 95 °C for 30 s, annealing at 55 °C for 40 s, and extension at 70 °C for 60 s for 40 cycles. GAPDH was used as an internal control. In each experiment, 3 parallel sub-wells were set up to analyze gene expression, and the difference (Ct value) between parallel wells, which should be <1, was quantified relative to the control group value by Equation (2) ^-^ΔΔCt [84].

### 4.7. Western Blot Analysis

The cells in each group (Control, Iso, Iso + CGA 10 µM, Iso + CGA 50 µM, Iso + CGA 100 µM, Iso + CGA 150 µM, IM-12 + Iso + CGA 150 µM) were treated with a cocktail of protease and phosphatase inhibitors, and then their protein concentration was assessed using a bicinchoninic acid disodium (BCA) protein quantification kit (P0010) according to the instructions of the manufacturer. All samples were subsequently adjusted to the same volume with 2 × 4% sodium dodecyl sulfate (SDS); then, the sample buffer was added, and the samples were boiled for 10 min. Equal amounts of proteins were separated by 10% SDS-PAGE electrophoresis. The fractionated proteins were transferred to polyvinylidene fluoride (PVDF) (IPVH00010) membranes by electrophoresis at 120 V for 75 min. The membranes were blocked with 5% nonfat dry milk for 90 min and incubated with the appropriate concentrations of primary antibodies (1:1000) overnight at 4 °C. The antibodies used were as follows: anti-LRP6 (1:1000), -β-catenin (1:1000), -GAPDH (1:5000), -c-Myc (1:1000), -GSK-3β (1:1000), -p-GSK-3β (1:1000), -ANP (1:1000), and -BNP (1:1000). Then, the membranes were washed 3 times with PBS–Tween-20 and further incubated with HRP-conjugated secondary antibodies (1:5000) for 60 min at room temperature. GAPDH protein level was assessed as an internal reference by the same procedure. The incubated membranes were visualized by a chemiluminescence imaging system, and the blots were analyzed using ImageJ (version 1.83; National Institutes of Health) to semi-quantify the protein expression levels.

### 4.8. Data Acquisition from the GEO Database

The mRNA expression data characterizing hypertrophic cardiomyopathy (HCM) were obtained from the Gene Expression Omnibus (GEO) database, accessible at https://www.ncbi.nlm.nih.gov/gds (accessed on 5 November 2023). A total of 106 HCM samples and 39 control cardiac tissue samples were retrieved from the GSE36961 dataset for differential expression analysis. Additionally, this dataset was chosen for weighted gene co-expression network analysis. Subsequently, the microarray dataset GSE85871 was also obtained from the Gene Expression Omnibus database to investigate the impact of CGA on cells, and the analysis of the differentially expressed genes (DEGs) was conducted.

### 4.9. Data Preprocessing and Differential Expression Analysis

Multiple intergroup comparisons were conducted using Sangerbox 3.0 (http://vip.sangerbox.com/home.html, accessed on 6 November 2023), an openly accessible online data analysis and visualization platform. The initial step involved normalizing the expression of mRNA in the GSE36961 and GSE85871 datasets (AD and PD). Subsequently, volcano plots and heatmaps were created to visualize the differentially expressed genes (DEGs). DEGs demonstrating an absolute fold change of ≥1.5 and a *p*-value < 0.05 were considered significantly different statistically in terms of their expression.

### 4.10. Weighted Gene Co-Expression Network Analysis (WGCNA)

To identify modules of closely correlated genes and hub genes, this study employed the WGCNA [86] approach to construct a scale-free co-expression network using the GSE36961 dataset. The initial step involved Pearson correlation-based hierarchical clustering to group all genes and samples. Subsequently, the soft threshold power value was determined to establish a co-expression network, ensuring its adherence to a scale-free structure. The adjacency matrices were then transformed into a topological overlap matrix to identify gene modules. Furthermore, similar modules were merged and clustered together. Finally, module–trait relationship diagrams were generated, and gene lists associated with each module were obtained.

### 4.11. Identification of Wnt-Related Genes Affected by CGA in HCM-Affected Cells and Functional Enrichment Analysis

To obtain the Wnt signaling pathway-related genes targeted by CGA in HCM-affected cells, a Venn analysis was performed on the DEGs associated with CGA treatment, Wnt signaling pathway-related genes, the HCM-associated DEGs, and hub genes in WGCNA via a free online platform (https://www.bioinformatics.com.cn , accessed on 8 November 2023). Then, the Metascape database (https://metascape.org/gp/index.html, accessed on 8 November 2023) was used to perform functional enrichment analysis on the above intersection targets [87].

### 4.12. Statistical Analysis

All experimental data collected were analyzed with the GraphPad Prism 9.0 software (La Jolla, CA, USA), and the data are expressed as mean ± standard error. One-way ANOVA and Student’s t-test were applied to discriminate significant differences among the experimental groups; *p* < 0.05 indicated a statistically significant difference.

## 5. Conclusions

In conclusion, our study demonstrated that pretreatment of AC16 cells with CGA attenuated Iso-induced CH. The underlying mechanism appeared to be related to the inhibition of the regulation of protein expression in the Wnt/β-catenin signaling pathway. These results suggest that CGA may be a potential drug for the treatment of CH. These findings suggest that CGA may play an important role in the inhibition of the Wnt signaling pathway, which is involved in the regulation of the development of CH and provide a new direction for its clinical development.

## Figures and Tables

**Table 1:** qPCR primer sequences [85].

Primer Name	Primer Sequence
ANP	Forward primer, 5′-CAGCAAGCAGTGGATTGCTCCT-3′ Reverse primer, 5′-TCTGCGTTGGACACGGCATTGT-3′
BNP	Forward primer, 5′-TGGAAACGTCCGGGTTACAGGA-3′ Reverse primer, 5′-TCCGGTCCATCTTCCTCCCAAA-3′
β-MHC	Forward primer, 5′-GGGCAAAGGCAAGGCCAAGAAA-3′ Reverse primer, 5′-ATGGGTGGAGCGCAAGTTGGTCA-3′
GAPDH	Forward primer, 5′-GGAGCGAGATCCCTCCAAAAT-3′ Reverse primer, 5′-GGCTGTTGTCATACTTCTCATGG-3′

ANP, atrial natriuretic peptide, BNP, brain natriuretic peptide, β-MHC, β-myosin heavy chain.

## Data Availability

The datasets generated and/or analyzed during the current study are publicly available.
